# Histological Characterization of Human Breast Implant Capsules

**DOI:** 10.1007/s00266-014-0439-7

**Published:** 2015-03-06

**Authors:** Janine M. Bui, TracyAnn Perry, Cindy D. Ren, Barbara Nofrey, Steven Teitelbaum, Dennis E. Van Epps

**Affiliations:** 1 Allergan, Inc., 2525 Dupont, Irvine, CA 92612 USA; 2 Division of Plastic and Reconstructive Surgery, David Geffen School of Medicine at UCLA, Los Angeles, CA USA

**Keywords:** Breast implant, Capsular contracture, Collagen fiber alignment, α-Smooth muscle actin, Myofibroblast, Baker score

## Abstract

**Background:**

This study investigated the relationships between histomorphological aspects of breast capsules, including capsule thickness, collagen fiber alignment, the presence of α-smooth muscle actin (α-SMA)–positive myofibroblasts, and clinical observations of capsular contracture.

**Methods:**

Breast capsule samples were collected at the time of implant removal in patients undergoing breast implant replacement or revision surgery. Capsular contracture was scored preoperatively using the Baker scale. Histological analysis included hematoxylin and eosin staining, quantitative analysis of capsule thickness, collagen fiber alignment, and immunohistochemical evaluation for α-SMA and CD68.

**Results:**

Forty-nine samples were harvested from 41 patients. A large variation in histomorphology was observed between samples, including differences in cellularity, fiber density and organization, and overall structure. Baker I capsules were significantly thinner than Baker II, III, and IV capsules. Capsule thickness positively correlated with implantation time for all capsules and for contracted capsules (Baker III and IV). Contracted capsules had significantly greater collagen fiber alignment and α-SMA–positive immunoreactivity than uncontracted capsules (Baker I and II). Capsules from textured implants had significantly less α-SMA–positive immunoreactivity than capsules from smooth implants.

**Conclusion:**

The histomorphological diversity observed between the breast capsules highlights the challenges of identifying mechanistic trends in capsular contracture. Our findings support the role of increasing capsule thickness and collagen fiber alignment, and the presence of contractile myofibroblasts in the development of contracture. These changes in capsule structure may be directly related to palpation stiffness considered in the Baker score. Approaches to disrupt these processes may aid in decreasing capsular contracture rates.

**Level of Evidence III:**

This journal requires that authors assign a level of evidence to each article. For a full description of these Evidence-Based Medicine ratings, please refer to the Table of Contents or the online Instructions to Authors www.springer.com/00266.

## Introduction

Placement of a breast implant initiates a foreign body response and ultimately results in the formation of a collagenous capsule. One of the most common complications associated with the presence of this collagenous capsule is capsular contracture, which can result in pain, discomfort, and distortion of the implant and the breast. The frequency of the clinical manifestation of contracture varies dramatically in patients and may be influenced by a number of exogenous factors, including surgical technique, pocket fit, bleeding, trauma, implant fill, implant surface, incision location, placement relative to pectoralis major muscle, infection or biofilm formation, breast reconstruction, radiation therapy, implant compromise including gel migration [1–3], and others. With the many factors influencing capsular contracture, identifying a relationship between histological features of capsules and clinical presentation of contracture may shed light on a common underlying etiology or mechanism of contracture.

The foreign body response to an implanted device is initiated by an inflammatory reaction followed by recruitment of fibroblasts, which lay down collagen fibers, and contractile myofibroblasts, which generate the force generally associated with contracture. At some point, myofibroblasts undergo apoptosis and contractile forces may cease, whereas the collagen structure remains. Capsular contracture results when the normal healing process fails or when a pathological change is initiated by tissue trauma or an exogenous trigger. The continued activity of fibroblasts and myofibroblasts in a breast capsule may result in highly aligned fibers and a rigid collagen capsule [4]. Highly aligned collagen fibers would theoretically be associated with a greater force of contracture when myofibroblasts are stimulated to contract along uninterrupted parallel fibers [5].

A number of surgical and prophylactic approaches have been used to reduce the incidence of capsular contracture, including surface texturing of the device, submuscular implantation, and reduction of bacterial contamination through nipple shields and antibiotic washes [6, 7]. The current study was directed at elucidating the relationship between capsular contracture, as measured by Baker score, and histological features of the capsules, including the presence of myofibroblasts and quantitative assessment of collagen fiber alignment and capsule thickness.

## Materials and Methods

### Clinical Profile

Forty-nine tissue samples were harvested at the time of implant removal from the anterior side of capsules surrounding breast implants from 41 female patients undergoing breast implant replacement or revision surgery. Specimens and clinical data were collected between 2009 and 2011 by Dr. Steven Teitelbaum after informed, written consent was obtained in accordance with the Declaration of Helsinki.

**Fig. 1:**
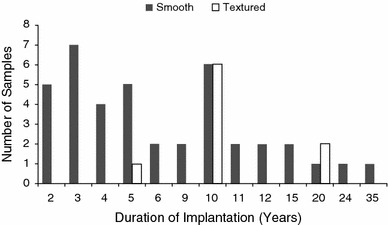
Summary of patient implants with respect to time from implantation to explant. Duration for smooth implants (*n* = 40) ranged from 2 to 35 years with an average of 7.9 years, whereas duration for textured implants (*n* = 9) ranged from 5 to 20 years with an average of 11.7 years. Overall duration averaged 8.6 years for all implants

Clinical capsular contracture was scored preoperatively using standard Baker score criteria. Baker scores were determined by a single, well-experienced physician (Dr. Steven Teitelbaum) using standard scoring criteria to minimize the potential for interphysician variability. The Baker assessment was done blinded to the data subsequently generated and all aspects of the technical assessment were done in the absence of knowledge of the Baker score, so that results in either direction were not influenced by the clinical or laboratory results. Tissue samples were collected as part of routine pathology assessment of capsular tissue. Residual tissue from the pathology assessment was utilized in this study. Inclusion criteria included any female patient presenting to the practice of Dr. Steven Teitelbaum for implant revision. Patients with implant rupture were excluded from this study. Although Baker II capsules are considered to be slightly contracted, for this dataset the designation of an “uncontracted” capsule refers to a Baker score of I or II, and the designation of a “contracted” capsule refers to a Baker score of III or IV. Patient profile and implantation duration information are summarized in Table 1 and Fig. 1.

**Table 1:** Patient data

	Uncontracted		Contracted		Total
	Baker I	Baker II	Baker III	Baker IV	
*All implants*, *n (%)*	6 (12.2)	12 (24.5)	28 (57.1)	3 (6.1)	49 (100.0)
*Duration (implant to explant [years]), mean ± SD*	6.1 ± 4.9	8.6 ± 4.9	9.9 ± 7.6	4.0 ± 1.7	8.7 ± 6.6
*Implant surface*, *n (%)*					
Biocell^®^	0	3 (6.1)	3 (6.1)	0	6 (12.2)
Siltex^®^	1 (2.0)	1 (2.0)	1 (2.0)	0	3 (6.1)
Smooth	5 (10.2)	8 (16.3)	24 (49.0)	3 (6.1)	40 (81.6)
*Implant placement*, *n (%)*					
Dual plane	0	1 (2.0)	3 (6.1)	0	4 (8.2)
Subglandular	3 (6.1)	6 (12.2)	9 (18.4)	0	18 (36.7)
Submuscular	3 (6.1)	5 (10.2)	16 (32.7)	3 (6.1)	27 (55.1)
*Reason for implantation*, *n (%)*					
Reconstruction	0	0	1 (2.0)	0	1 (2.0)
Augmentation	6 (12.2)	12 (24.5)	27 (55.1)	3 (6.1)	48 (98.0)
*Reason for explantation*, *n (%)*					
Contracture	0	4 (8.2)	24 (49.0)	3 (6.1)	31 (63.3)
Revision surgery	0	0	2 (4.1)	0	2 (4.1)
Complication with other breast	2 (4.1)	6 (12.2)	2 (4.1)	0	10 (20.4)
Size change or implant removal	4 (8.2)	2 (4.1)	0	0	6 (12.2)

### Histology and Immunohistochemistry

Tissue samples were fixed in 10 % neutral-buffered formalin, then processed and embedded in paraffin. Sections were cut at 5 μm for hematoxylin and eosin (Richard-Allan Scientific, Kalamazoo, MI, USA) staining and immunohistochemistry.

Immunohistochemical evaluation was performed using monoclonal antibodies specific for α-smooth muscle actin (α-SMA), an indicator of myofibroblast presence (Clone 1A4, DAKO, Glostrup, Denmark) and for CD68 (Clone KP1, DAKO, Glostrup, Denmark [antibody recognizes a 110-kDa glycoprotein expressed on monocytes and macrophages]). All immunohistochemistry was performed using the EnVision™ FLEX High pH visualization system (DAKO, Glostrup, Denmark).

**Fig. 2:**
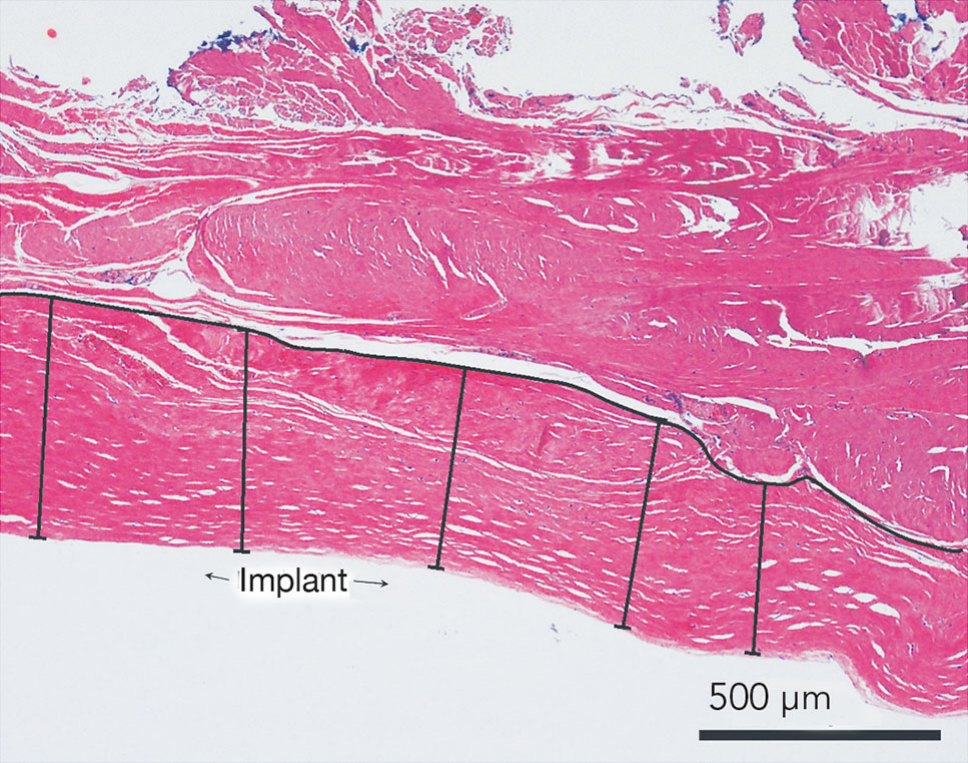
Measurement of capsular thickness. Capsular thickness was measured by drawing a *line* to delineate the interface between capsule and surrounding tissue where the capsule was defined as the layer of collagenous tissue closest to the implant. Five measurements were taken between the delineating *line* and the edge of the tissue

**Fig. 3:**
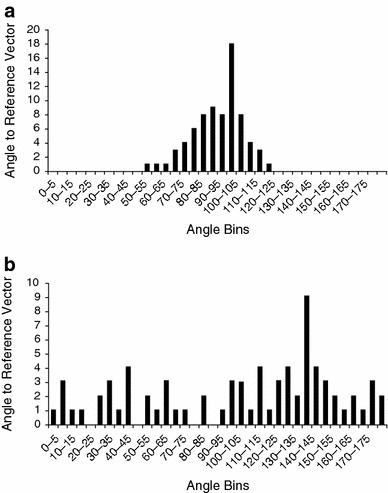
**a** Distribution of vectors for a highly aligned capsule with a standard deviation of 13.30 and **b** a highly unaligned capsule with a standard deviation of 50.21. The distribution of vector angles is representative of fiber alignment and is quantitated by the standard deviation of vectors. If all fibers are parallel, all angles will be either 0° or 180° and the standard deviation of vector angles would be 0. If none of the fibers are parallel, angles will be equally distributed across all measures from 0° to 180°

**Fig. 4:**
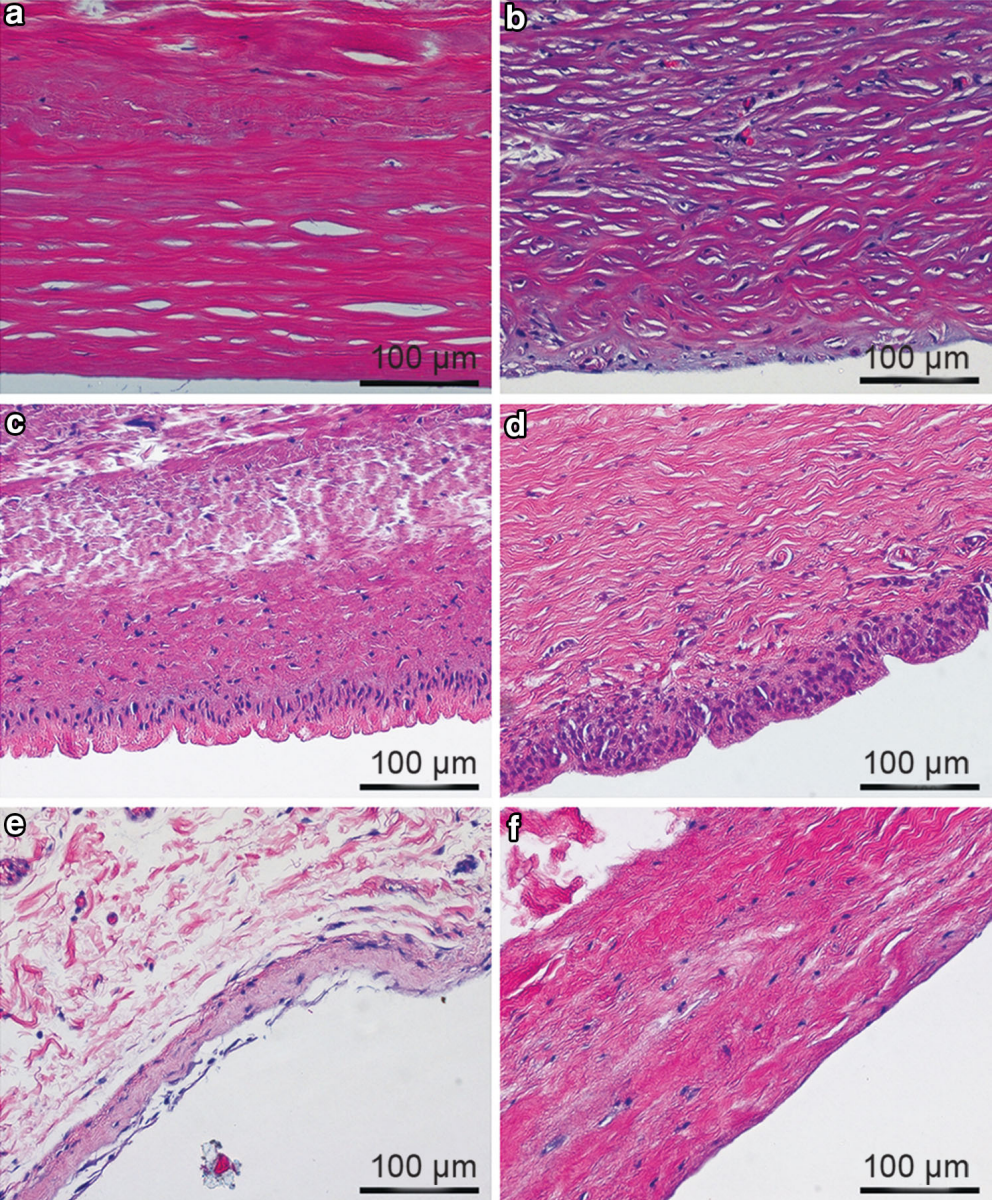
Hematoxylin and eosin staining of human capsules (magnification ×20, *scale bar* 100 µm). All images are oriented with the implant-tissue interface in the *lower portion* of the image. **a** Baker IV contracted capsule with low cellularity and thick dense bands of highly aligned fibers taken from a smooth silicone implant after 3 years of submuscular implantation. **b** Baker IV contracted capsule with increased cellularity and thick dense bands of highly aligned fibers taken from a smooth silicone implant after 3 years of submuscular implantation. **c** Baker II capsule with morphology consistent with synovial metaplasia taken from a textured saline implant after 10 years of dual plane implantation. **d** Baker III capsule with morphology consistent with synovial metaplasia taken from a smooth silicone implant after 15 years of submuscular implantation. **e** Thin Baker I capsule with loosely arranged fibers taken from a smooth saline implant after 3 years of submuscular implantation. **f** Baker I capsule with low cellularity and loosely arranged fibers taken from a smooth saline implant after 12 years of subglandular implantation

General characteristics of the histopathology of implant capsules with different Baker scores were assessed visually by review of hematoxylin and eosin-stained capsule samples. Capsules were classified into four categories: (1) dense collagen, acellular or low cellular content (example Fig. 4a), (2) dense collagen, moderate to high cellular content (example Fig. 4b), (3) synovial metaplasia (example Fig. 4c, d), or (4) loosely packed collagen (example Fig. 4e, f).

### Image Analysis

Sections were imaged at ×4 and ×20 magnifications and analyzed using Nikon NIS Elements Advanced Research software (Nikon, Melville, NY, USA).

Capsular thickness was measured from five evenly spaced measurements of the capsule on a representative ×4 magnification image as shown in Fig. 2. A capsule was defined as the collagen fiber layer of tissue closest to the implant surface.

Alignment of capsular collagen fibers was assessed by vector analysis measuring the extent to which the fibers were parallel to the surface of the implant. A reference vector was drawn parallel to the tissue-device interface on a ×20 magnification image of a hematoxylin and eosin-stained section of the tissue. Twenty-five additional vectors were drawn along individual collagen fibers and the angles relative to the reference vector were measured. This was repeated for a total of three images and 75 vector measurements per sample. Vector angles were normalized to the surface of the implant. The standard deviation of the normalized vector angles was used as a measure of alignment, in which a highly aligned sample has a lower standard deviation of fiber angles (Fig. 3a), and a highly unaligned sample has a higher standard deviation of fiber angles (Fig. 3b).

To provide a rigorous exclusion of non-specific antibody binding, immunostained samples were considered positive for α-SMA if elongated and fibrous staining was visible in ≥10 % of the capsule layer proximal to the implant. CD68-stained samples were considered positive if cytoplasmic staining was observed in >10 cells per ×20 field.

### Statistical Analysis

Statistical analysis for the comparison of capsule thickness and fiber alignment by Baker score was performed using a Kruskal–Wallis test. For *p* values of less than 0.05, a Mann–Whitney *U* test was used to determine the significance of the difference between the pairs of Baker score groups. All other pairwise comparisons were performed using the Mann–Whitney test. All statistical analyses for immunopositive staining of α-SMA and CD68 were performed using a *χ* ^2^ test. Linear regression analysis was used to assess the impact of implantation time. A *p* value of less than 0.05 was considered significant. All numerical data for thickness and fiber alignment are presented as a mean ± standard deviation unless otherwise noted. Outliers were included in all statistical analyses except linear regression analysis. All statistical analyses were performed using Minitab 15 Statistical Software (Minitab Inc., State College, PA, USA).

In this study, the population was acquired based on clinical need for breast revision surgery at a single clinical site and as a result it does not represent a homogeneous sample. Variables include implant type (smooth or textured), duration of implant placement, plane of implantation, and reason for explantation. Details of the patient population are summarized in Table 1. Statistical analyses accounted for the heterogeneity of the population and whenever possible (based on number of events), patient subsets were independently analyzed. Because the common characteristic in all patients was implant revision in the absence of implant rupture, these patients were grouped in the overall analysis of Baker score and histomorphological assessment. All patients underwent augmentation revision with the exception of one patient who underwent reconstruction revision. The patient who underwent reconstruction revision had the longest time from implantation to revision (35 years).

## Results

### Capsule Architecture and Morphology

A large variation in histomorphology was observed between samples, including variations in cellularity, fiber density, fiber organization, vascularization, and overall structure. Capsules were generally found to have low cellularity, although there was evidence of regions of increased or concentrated cellularity in some cases at or near the capsule-implant interface. Multiple layers of fibers of differing fiber density and alignment were identified in a number of samples, whereas other capsules were composed of a single-collagen layer of variable density. In general, the capsule region adjacent to the implant lacked vascularization, although vascularization throughout the entire capsule was evident in a small number of samples. Contracted capsules were found to contain thick, dense bands of highly aligned fibers (Fig. 4a, b, d), whereas uncontracted capsules were composed of thin, loosely arranged, multidirectional, string-like fibers (Fig. 4e, f). Morphology consistent with synovial metaplasia was observed in some samples and was characterized by a layer of synovial-like cells arranged in a palisaded manner at the capsule-implant interface (Fig. 4c, d).

### Capsular Thickness

**Fig. 5:**
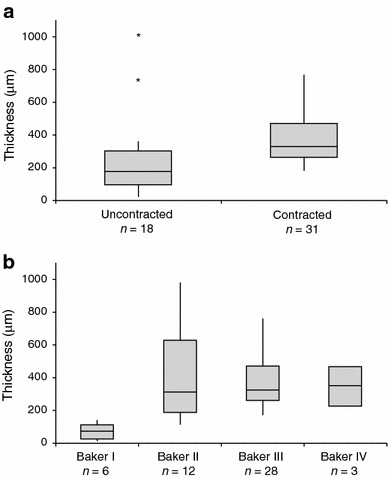
Box plot of capsular thickness by level of contracture. The whiskers represent the minimum and maximum values. The *upper* and *lower* edges of the *box* represent the 25th and 75th percentile, respectively, and the band represents the median. **a** Contracted capsules (mean = 389.8 µm) are significantly thicker than uncontracted capsules (mean = 285.3 µm; *p* = 0.0111). Three statistical outliers were identified in the uncontracted group. Outliers included a Baker II capsule from a smooth device that had been implanted for 10 years (thickness = 996 µm), and two Baker II capsules from textured devices that had been implanted for 10 years (thickness = 736 and 723 µm). **b** Baker I capsules are significantly thinner (mean = 91.5 µm) than Baker II (mean = 408.6 µm; *p* = 0.0012), III (mean = 393.4 µm; *p* = 0.0002), and IV capsules (mean = 355.4 µm; *p* = 0.0282). *Represents statistical outliers

**Fig. 6:**
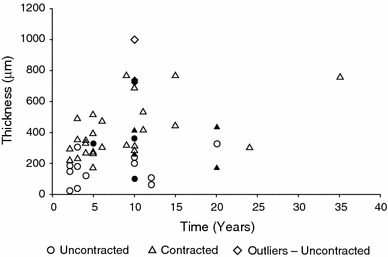
Capsular thickness was positively correlated with duration of implantation for all capsules (*R* ^2^ = 0.151; *p* = 0.0076) and for contracted capsules (*R* ^2^ = 0.159; *p* = 0.026), but not for uncontracted capsules (*p* = 0.296). *Solid* data points are from textured implants and *open* data points are from smooth implants. Statistical outliers were only identified in the uncontracted group and were not included in regression analysis. The sample identified at 35 years represents the one patient with breast reconstruction and revision

Capsular thickness ranged from 21 to 996 µm, with a mean of 351.4 ± 215.4 µm. There was no significant difference (*p* = 0.4777) in capsule thickness between smooth (mean = 342.5 ± 216.0 µm, *n* = 40) and textured implants (mean = 391.0 ± 220.7 µm, *n* = 9), although the number of textured implants was limited and included both Siltex^®^ and Biocell^®^ devices (no manufacturer information was available for smooth implants). Uncontracted capsules (Baker I and II, mean = 285.3 ± 270.3 µm) were significantly thinner (*p* = 0.0111) than contracted capsules (Baker III and IV, mean = 389.8 ± 169.4 µm, Table 2; Fig. 5a). No significant difference in thickness was found between Baker II, III, and IV capsules (*p* = 0.716, Fig. 5b). However, Baker I capsules (mean = 91.5 ± 30.3 µm) were found to be significantly thinner than Baker II (mean = 408.6 ± 28.9 µm; *p* = 0.0012), III (mean = 393.4 ± 24.5 µm; *p* = 0.0002), and IV capsules (mean = 355.4 ± 17.9 µm; *p* = 0.0282). No significant difference in thickness was found based on plane of implantation (*p* = 0.152). Capsule thickness was positively correlated with implantation time for all capsules (*R* ^2^ = 0.151; *p* = 0.0076; Fig. 6) and for contracted capsules alone (*R* ^2^ = 0.159; *p* = 0.026), but not for uncontracted capsules alone (*p* = 0.296).

**Table 2:** Summary of uncontracted versus contracted analysis of capsules

Capsule characteristic	Uncontracted		Contracted		Uncontracted	Contracted	*p* value^a^
	Baker I	Baker II	Baker III	Baker IV			
*Thickness (µm), mean ± SD*	91.5 ± 30.3	408.6 ± 28.9	393.4 ± 24.5	355.4 ± 17.9	285.3 ± 270.3	389.8 ± 169.4	0.0111
*Collagen fiber alignment (angle SD), mean ± SD*	30.3 ± 5.6	28.9 ± 9.5	24.5 ± 8.3	17.9 ± 3.1	29.4 ± 8.1	23.8 ± 8.2	0.0068
*α-SMA, % of positive samples*	17	9	39	33	12	39	0.049
*Histopathology of capsule, % of samples*							
Dense collagen							
Acellular^b^	43	55	75	67	50	81	0.001
Cellular^c^	14	18	14	33	17	10	NS
Synovial metaplasia	29	9	7	0	17	6	NS
Loosely packed collagen	43	27	11	0	33	10	NS

*NS* not significant

^a^ *p* value for comparison of uncontracted (Baker I and II) versus contracted (Baker III and IV)

^b^Example shown in Fig. 4a

^c^Example shown in Fig. 4b

^d^Example shown in Fig. 4c, d

^e^Example shown in Fig. 4e, f

### Collagen Fiber Alignment

**Fig. 7:**
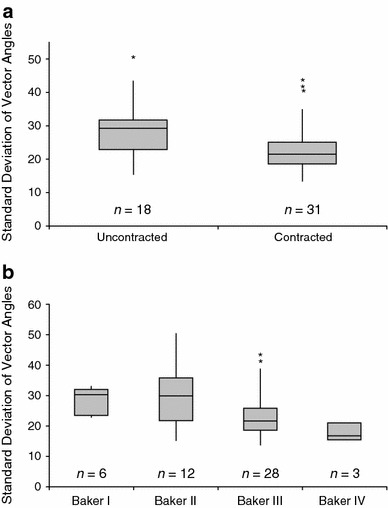
**a** *Box* plot of collagen fiber alignment by level of contracture. The whiskers represent the minimum and maximum values. The *upper* and *lower* edges of the box represent the 25th and 75th percentile, respectively, and the band represents the median. Contracted capsules (mean = 23.8) had fibers that were significantly more aligned than uncontracted capsules (mean = 29.4; *p* = 0.0068). **b** Fiber alignment increased with increasing Baker score (mean Baker scores: I = 30.3, II = 28.9, III = 24.5, and IV = 17.9). One outlier capsule was identified in the Baker II/uncontracted group from a textured device that had been implanted for 10 years (SD = 50.2). Three outliers were identified in the Baker III/contracted group, including a capsule from a textured device that had been implanted for 10 years (SD = 43.3), a capsule from a smooth device that had been implanted for 9 years (SD = 41.1), and a capsule from a smooth device that had been implanted for 2 years (SD = 39.32). *Represents statistical outliers

The standard deviation of the vector angles of collagen fibers with respect to the implant surface was used as a measure of alignment and ranged from 13.3 to 50.2 (mean = 25.9 ± 8.5), in which a lower standard deviation indicates greater alignment. No significant difference (*p* = 0.1631) in fiber alignment was observed between capsules from smooth (mean = 24.8 ± 7.8) and textured implants (mean = 30.7 ± 10.1), although this may simply reflect the lower number of textured implants (*n* = 9) analyzed as well as the mixture of both Siltex^®^ and Biocell^®^ devices. Contracted capsules (mean = 23.8 ± 8.2) showed significantly greater fiber alignment (*p* = 0.0068) than uncontracted capsules (mean = 29.4 ± 8.1; Table 2; Fig. 7a). Baker I capsules (mean = 30.3 ± 5.6) were found to be significantly less aligned than Baker III (mean = 24.5 ± 8.3; *p* = 0.0494) and Baker IV capsules (mean = 17.9 ± 3.1; *p* = 0.0282), and Baker II capsules (mean = 28.9 ± 9.5) were found to be significantly less aligned than Baker IV capsules (*p* = 0.0364) as shown in Fig. 7b. No significant difference in fiber alignment was found based on plane of implantation (*p* = 0.418). Fiber alignment was not correlated with time from implantation.

### Myofibroblasts (α-SMA–Positive Immunoreactive Staining)

**Fig. 8:**
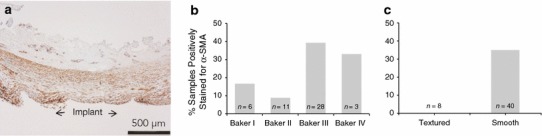
α-Smooth muscle actin (α-SMA) staining of human capsules (magnification ×4, *scale bar* 500 µm). **a** Representative α-SMA–positive staining where myofibroblasts can be seen localized to the tissue-device interface. **b** Percentage of capsules α-SMA–positive for myofibroblasts by Baker score. **c** Percentage of capsules α-SMA–positive for myofibroblasts by implant surface

Myofibroblasts were identified using immunohistochemical staining for α-SMA, and, when present, were localized near the tissue-device interface (Fig. 8a). One Baker II textured sample was excluded from the analysis due to insufficient tissue adherence to the slide. A significant difference (*p* = 0.049) in α-SMA–positive immunoreactivity was found based on contracture state, in which 39 % of contracted capsules and 12 % of uncontracted capsules were positive for α-SMA (Table 2). A lower percentage of Baker I (17 %) and Baker II capsules (9 %) were positive for α-SMA compared with Baker III (39 %) and Baker IV capsules (33 %; Fig. 8b). All capsules from textured implants were found to be negative for α-SMA, whereas 35 % of capsules from smooth implants stained positive, which was a statistically significant difference (*p* = 0.047; Fig. 8c). The number of positive samples in the Baker I, II, and IV groups were too small to allow for statistical analysis. No significant difference (*p* = 0.602) in α-SMA–positive immunoreactivity was identified based on plane of implantation.

### Summary of Capsule Histopathology

The majority of Baker III and IV capsules exhibited a dense acellular or low cellular content morphology, whereas Baker I and II capsules exhibited predominantly more loosely packed collagen. It is also interesting to note that synovial metaplasia was more common in Baker I and II capsules compared with Baker III and IV capsules.

### Macrophages (CD68-Positive Immunoreactive Staining)

Macrophages were identified using immunohistochemical staining for CD68. No significant difference in CD68-positive immunoreactivity was observed based on contracture status (*p* = 0.737) or duration of implantation (*p* = 0.5001). Analysis of CD68-positive immunoreactivity was not possible by plane of implantation or Baker score due to limited sample groups. All textured implants and 81 % of smooth implants were positive for CD68; however, this difference was not statistically significant (*p* = 0.174).

## Discussion

The dataset in this study included 49 capsule samples with Baker classification scores I through IV, and duration of implant ranging from 2 to 35 years. Capsule tissues from both smooth and textured implants were compared, although the majority of the samples were derived from smooth implants. All Baker IV capsules were from smooth implants. Due to the small number of samples derived from textured devices (*n* = 9) and the inclusion of two different types of surface texture (Siltex^®^ and Biocell^®^), no conclusions could be drawn with respect to the impact of each type of textured surface. Although the population varies in age, implant type, reason for revision, and time to revision, several common characteristics relating to increased Baker score and capsule structure exist, including capsule thickness, collagen structure, and staining of α-SMA for myofibroblasts.

The alignment of collagen fibers was measured quantitatively using a mathematically rigorous approach. Published literature suggests that collagen fiber alignment is routinely assessed in a qualitative manner by classification of fibers as either aligned or unaligned, or by a descriptive narrative of fiber orientation [8–13]. Figure 3 shows the distribution of vector angles for the most aligned and the least aligned samples in the dataset. In this study, fibers were found to be progressively more aligned with increasing Baker score. A statistically significant difference in alignment was demonstrated between capsules when grouped as uncontracted (Baker I and II) and contracted (Baker III and IV), as well as when capsules were grouped by individual Baker scores. This supports the theory that alignment of collagen fibers is a key feature in capsular contracture, and suggests that disruption of collagen fiber alignment may decrease the incidence and severity of capsular contracture [5]. Although capsules from textured implants were less aligned than capsules from smooth implants, this difference failed to reach statistical significance, likely a result of the small number of samples from textured implants and the presence of two different types of textured surfaces. There was no correlation between fiber alignment and time from implantation for contracted or uncontracted samples.

The diversity of the sample population was reflected in the histomorphological variation in the capsule tissue, which showed large differences in degree of cellularity, fiber density and organization, vascularization, and gross overall structure. Although no definitive pathological identification was made, morphology consistent with synovial metaplasia was observed in several samples and has previously been hypothesized to be a response to mechanical stress. The presence of synovial metaplasia-like morphology is well documented [10, 13–16] and may serve a lubricating function between tissue and implant [17].

Capsule thickness was found to correlate significantly with contracture, in which Baker I capsules were found to be significantly thinner than Baker II, III, and IV capsules. This suggests that capsule thickening may contribute to the transition from an uncontracted Baker I capsule to the initial stages of Baker II contracture. Although the relationship between capsule thickness and contracture remains to be fully elucidated, several studies have shown that Baker III and IV capsules are thicker than Baker I and II capsules [18–20]. Thickness for all capsules and contracted capsules alone, but not for uncontracted capsules alone, was found to increase with time from implantation (Fig. 6), suggesting that fibroblasts continue to lay down collagen fibers long after implantation.

Myofibroblasts are contractile fibroblasts that play an active role in wound closure during healing and are commonly reported in capsule morphology [4, 21–23]. Appropriately stimulated fibroblasts initially develop into protomyofibroblasts, which have limited contractility, and then into differentiated myofibroblasts, which are capable of generating large contractile forces [21]. Immunopositive staining for α-SMA, a marker for differentiated myofibroblasts [24–26], demonstrated localization of myofibroblasts near the capsule-device interface, consistent with the findings of Hwang et al. [4]. A significantly higher percentage of contracted capsules as compared with uncontracted capsules were found to be immunopositive for myofibroblasts. This is consistent with the hypothesis that myofibroblasts play an active role in capsular contracture [4, 21].

Samples from textured implants were all found to be negative for myofibroblasts, suggesting that a textured surface influences capsular contracture by reducing the presence of myofibroblasts in the capsular tissue. Although the mechanism by which this reduction of myofibroblasts takes place has yet to be elucidated, the morphology of the three Baker IV capsules in this study may provide clues. Of the three capsules, only one was found to be immunopositive for α-SMA. The α-SMA–positive Baker IV capsule showed increased cellularity and vascularization and was histomorphologically distinct from the other two α-SMA–negative Baker IV capsules. Myofibroblasts are well documented to be present during the active period of wound healing but diminish as wounds progress to a more mature state [24]. It may be that the Baker IV capsules that did not show myofibroblast presence had progressed to a more mature state. In this case, a contractile force may be exhibited early by stimulated myofibroblasts resulting in contracture, which is then physically maintained by virtue of the deposition of a dense collagen capsule which retains the physically contracted state. The diminished presence of myofibroblasts and α-SMA staining in the presence of contracture may then be expected much like what has been observed in wound healing and scar formation where myofibroblasts undergo apoptosis in the later stages [24]. It remains to be determined if the lack of myofibroblasts in capsules from textured implants is due to a more rapid progression of the capsule to a mature state or due to a reduction in myofibroblast differentiation from fibroblasts.

Fibrocyte-stimulating cytokines released by inflammatory cells are known to play an important role in regulating fibroblasts and modulating collagen deposition during wound healing. CD68-positive immunoreactivity was used as a marker for inflammatory infiltration. Although Kamel et al. [9] have suggested an inverse relationship between CD68-positive macrophages and the degree of contracture, our results revealed no relationship with state of contracture or implant surface. These results suggest that the role of inflammation in capsule formation is decidedly more complex than the simple presence or absence of macrophages [27].

The nine samples from textured implants in this study were derived from two different manufacturers, with each texture having a unique microscopic surface structure and interaction with tissue [5, 28]. Due to the limited sample size, textured sample data were pooled as in previously published reports [4, 10, 13, 20, 29]. This may, in part, have contributed to the lack of robust effects of texture on capsule formation. Despite these pooled samples, a significant difference in the presence of α-SMA–positive myofibroblasts was identified between capsules from smooth and textured implants, indicating that myofibroblasts play an important role in the biological effect of texture on capsular contracture.

Baker score, although subjective, has been utilized as a common way to assess the status of breast implants and the degree of capsule contracture. One of the critical components of the Baker classification is the degree of firmness of the breast. A Baker I score is considered to be normally soft, Baker II is considered to be mildly or a little firm, Baker III is considered to be firm or moderately firm with a beginning of distortion, and Baker IV is considered to be firm and quite distorted in shape. The basis of these changes is reflected in this study in the histomorphological changes observed with increasing Baker score. Although the assessment of breast firmness may be quite variable between physicians and between patients with different size and shaped breasts, and a different skin and tissue coverage, the data presented here demonstrate common histologic changes that correlate with and potentially influence the degree of firmness. In particular, capsule thickness and collagen fiber orientation independent of time may be considered to affect firmness and Baker score. Furthermore, the increased frequency of α-SMA–positive capsules indicative of myofibroblast activation also supports an additional component of increased firmness, since myofibroblast activation is associated with contracture of scar tissue and capsules.

## Conclusion

The aim of this study was to investigate the nature of capsular contracture as it relates to collagen fiber alignment, capsule thickness, and the presence of α-SMA–positive myofibroblasts and CD68-positive macrophages. The histomorphological diversity observed in these capsules highlights the challenges of identifying mechanistic trends in capsular contracture, which may be influenced by the diversity of the patient population, the surgical procedure, and timing of the explant. Clinical studies controlling for many of these factors often include only relatively short time periods and frequently lack histological data. Despite the significant diversity of the sample population, this histological characterization of samples ranging from 2 to 35 years of implant duration demonstrated a positive quantitative association between collagen fiber alignment and Baker score, a positive quantitative association between capsule thickness and Baker score, as well as a correlation of α-SMA–positive myofibroblasts with contracture and implant surface texture. These findings indicate that the mechanism of capsule contracture and capsule stiffness involves both capsule thickening, which may increase over time, and alignment of collagen fibers as well as the presence of contractile myofibroblasts. These changes were common in spite of the diverse population and individually unique histological variations in capsule tissue from one patient to another.
